# Supplementary material for: The Construction and Exploration of a Comprehensive MicroRNA Centered Regulatory Network in Foxtail Millet (Setaria italica L.)
Source: Front Plant Sci. 2022 May 6;13:848474. doi: 10.3389/fpls.2022.848474 (PMC9121102; doi:10.3389/fpls.2022.848474)
Supplement: Supplementary file 3 [file Data_Sheet_3.zip › Sit-miR171a_Seita.7G293500.1_931_TPlot.pdf]

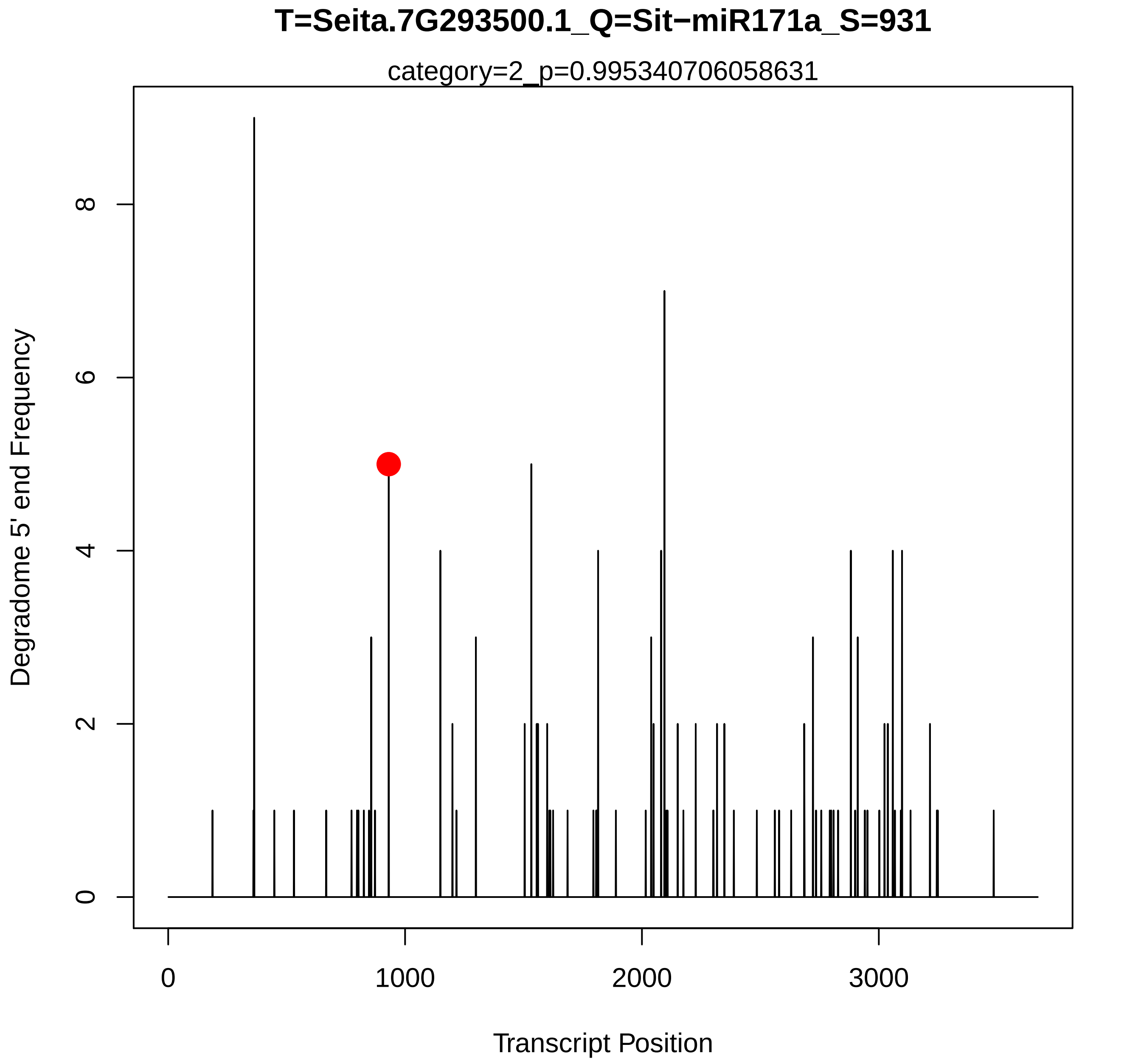
T=Seita.7G293500.1_Q=Sit−miR171a_S=931
category=2_p=0.995340706058631
Degradome 5' end Frequency
Transcript Position
0
2
4
6
8
0
1000
2000
3000
